# Diffusion mechanism in the sodium-ion battery material sodium cobaltate

**DOI:** 10.1038/s41598-018-21354-5

**Published:** 2018-02-16

**Authors:** T. J. Willis, D. G. Porter, D. J. Voneshen, S. Uthayakumar, F. Demmel, M. J. Gutmann, M. Roger, K. Refson, J. P. Goff

**Affiliations:** 1 Department of Physics, Royal Holloway, University of London, Egham, TW20 0EX UK; 2 ISIS Facility, Rutherford Appleton Laboratory, Chilton, Didcot, OX11 0QX UK; 3 Diamond Light Source, Harwell Science and Innovation Campus, Didcot, OX11 0DE UK; 4 Service de Physique de l’Etat Condensé, (CNRS/MIPPU/URA 2464), DSM/DRECAM/SPEC, CEA Saclay, P.C. 135, F-91191 Gif Sur Yvette, France

## Abstract

High performance batteries based on the movement of Li ions in Li_*x*_CoO_2_ have made possible a revolution in mobile electronic technology, from laptops to mobile phones. However, the scarcity of Li and the demand for energy storage for renewables has led to intense interest in Na-ion batteries, including structurally-related Na_*x*_CoO_2_. Here we have determined the diffusion mechanism for Na_0.8_CoO_2_ using diffuse x-ray scattering, quasi-elastic neutron scattering and *ab-initio* molecular dynamics simulations, and we find that the sodium ordering provides diffusion pathways and governs the diffusion rate. Above *T* ~ 290 K the so-called partially disordered stripe superstructure provides channels for quasi-1D diffusion, and melting of the sodium ordering leads to 2D superionic diffusion above *T* ~ 370 K. We obtain quantitative agreement between our microscopic study of the hopping mechanism and bulk self-diffusion measurements. Our approach can be applied widely to other Na- or Li-ion battery materials.

## Introduction

The high energy density of Li-ion batteries makes them ideal for energy storage for portable electronic devices and vehicles^1,2^. However, the increasing demand, and issues over supply of Li from remote or politically sensitive areas, have led to concerns over cost^3,4^. Furthermore, the enormous expansion of intermittent, renewable energy sources worldwide, will require energy storage systems for load levelling with a capacity orders of magnitude higher. The performance of Na-ion batteries is exceeded only by Li, and the high abundance and low cost of Na makes them an attractive alternative, particularly for medium to large-scale stationary energy storage, where weight is not the key issue^5–11^.

The commercial domination of the Li-ion battery market by layered oxides of the type Li_*x*_MO_2_ (M = Co, Mn, Ni) has led to the consideration of their Na analogues. De-intercalation and intercalation of Na in various layered Na_*x*_MO_2_ has been reported for a wide variety of transition metals (M = Ti^12^, V^13^, Cr^14^, Mn^15,16^, Fe^17^, Co^18,19^, Ni^20^) and, of these, Co and Mn are most viable for positive electrodes, with high capacity and good cycling properties. Li_*x*_CoO_2_ is the first and the most commercially successful layered oxide cathode, and we have chosen Na_*x*_CoO_2_ as a prototype for the study of diffusion in Na-ion battery materials.

The diffusion pathways and activation energies that govern ion transport within cathode materials control the rate at which a battery can be charged and discharged for high power applications. However, it is notoriously difficult to reconcile experimental determinations of diffusion rates measured by different probes with, for example, estimates of the diffusion coefficient of Li_*x*_CoO_2_ reported to vary over a wide range *D* ~ 10^–7^–10^–13^ cm^2^ s^−1^ in the literature^21^. Hopping events, as measured by microscopic probes, are often closely followed by a reverse hop, and do not contribute to bulk diffusion. For macroscopic measurements of diffusion using electrochemical cells, other factors such as device geometry and interface effects influence the measured result.

Quasi-elastic neutron scattering (QENS) is the only experimental technique that gives both temporal and microscopic spatial information and, therefore, it is possible to “see” experimentally the hopping of the diffusing ions. In a previous QENS study of Na_0.7_CoO_2_ the onset of Na dynamics was detected at *T* ~ 290 K^22^. However, in that study it was not possible to determine the wave-vector transfer, *Q*, dependence and, therefore, spatial information on the diffusion was limited. As a result it was not possible to distinguish between fast localized processes and translational diffusion. Here we have measured the *Q* dependence of the energy broadening of the QENS from Na_0.8_CoO_2_, and we are able to determine the microscopic diffusion mechanism. We explain the dramatic variation in diffusion rates as a function of composition and for different probes, and from the hopping distance and frequency, we obtain a diffusion rate that is in remarkably good agreement with bulk measurements.

## Results

### Sodium ordering

**Figure 1:**
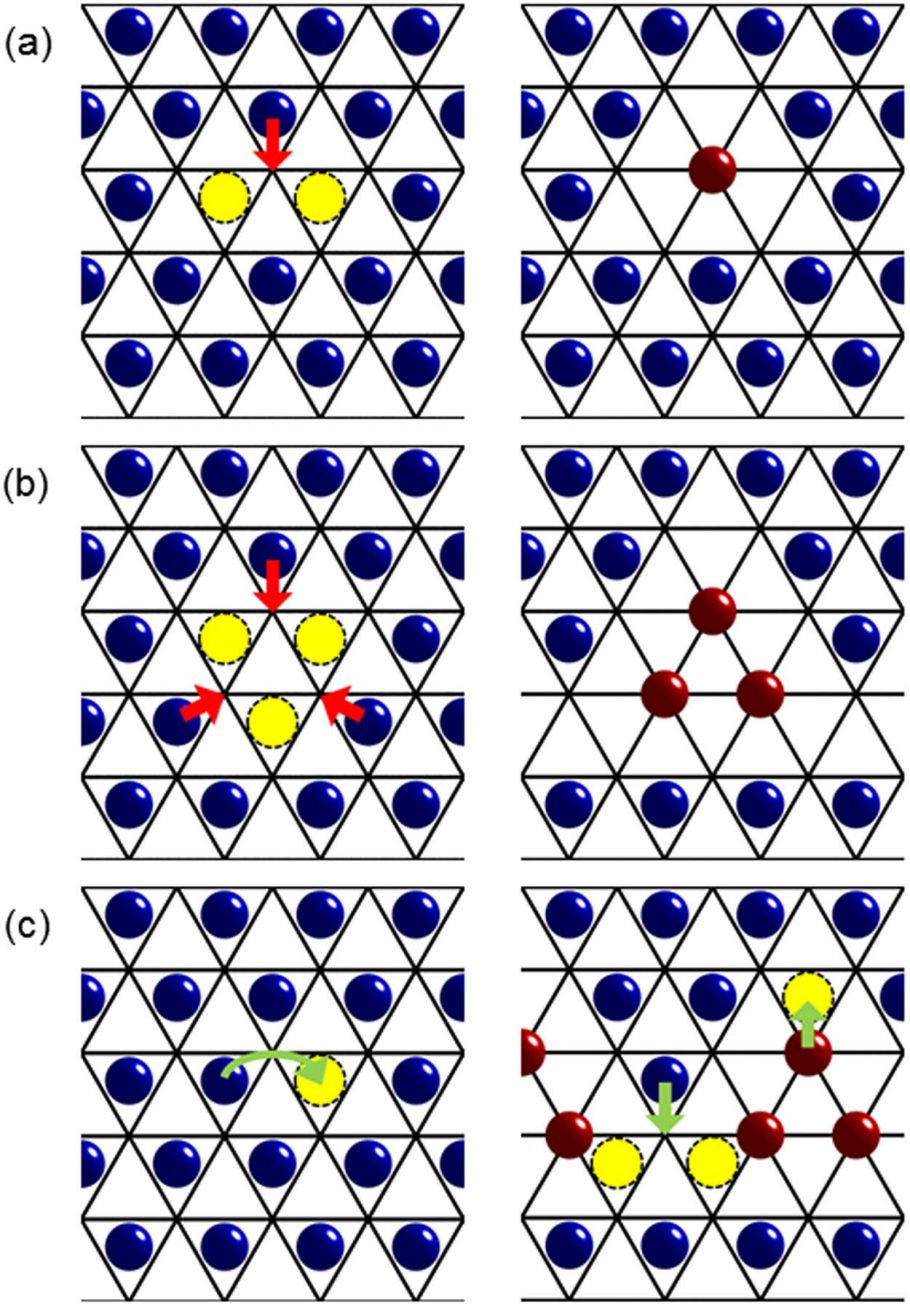
Multi-vacancy cluster formation and diffusion pathways in Na_*x*_CoO_2_. (**a**) For the di-vacancy cluster, left panel: there are two vacancies (yellow) on the underlying hexagonal lattice of 2*d* sites (blue), and in the right panel: one Na ion is promoted (red arrow) from a 2*d* site to a 2*b* site (red). (**b**) For the tri-vacancy cluster, there are three “real” vacancies and three Na ions are promoted from 2*d* to 2*b* sites. (**c**) Diffusion pathways (green arrows). Left panel: for isolated vacancies the hopping is between 2*d* sites, and in the right panel: in the presence of multi-vacancy clusters there is hopping between 2*b* and 2*d* sites.

Na_*x*_CoO_2_ exhibits a kaleidoscope of superstructures as a function of composition for *x* > 0.5 in which Na ions occupy two inequivalent Wyckoff sites, 2*b* and 2*d*, of the space group *P*6_3_/*mmc*^23–25^. Galvanostatic cycling reveals a series of potential steps associated with sodium reordering transitions that are reversible during the charge/discharge processes^19^. For Na_0.8_CoO_2_, static, long-range ordering of tri-vacancy clusters occurs below *T* ~ 290 K, in either a square or striped phase^25^. Figure 1 shows the formation of multi-vacancy clusters. At low temperature the ^23^Na NMR signal from Na_*x*_CoO_2_ can be explained in terms of the occupation of Na 2*b* and 2*d* sites in superstructures^26^. ^23^Na NMR studies of Na_0.75_CoO_2_ reveal anomalous mobility of specific Na sites above *T* ~ 180 K, and this is interpreted in terms of the rattling of ions in so-called “tri-vacancy clusters”^27^. These rattling modes in the square superstructure of Na_0.8_CoO_2_ have been measured by inelastic x-ray and neutron scattering supported by first-principles density-functional calculations^28^. The ^23^Na NMR response of Na_0.8_CoO_2_ above *T* ~ 290 K is similar to that previously observed in superionic conductors with planar Na layers^29^.

**Figure 2:**
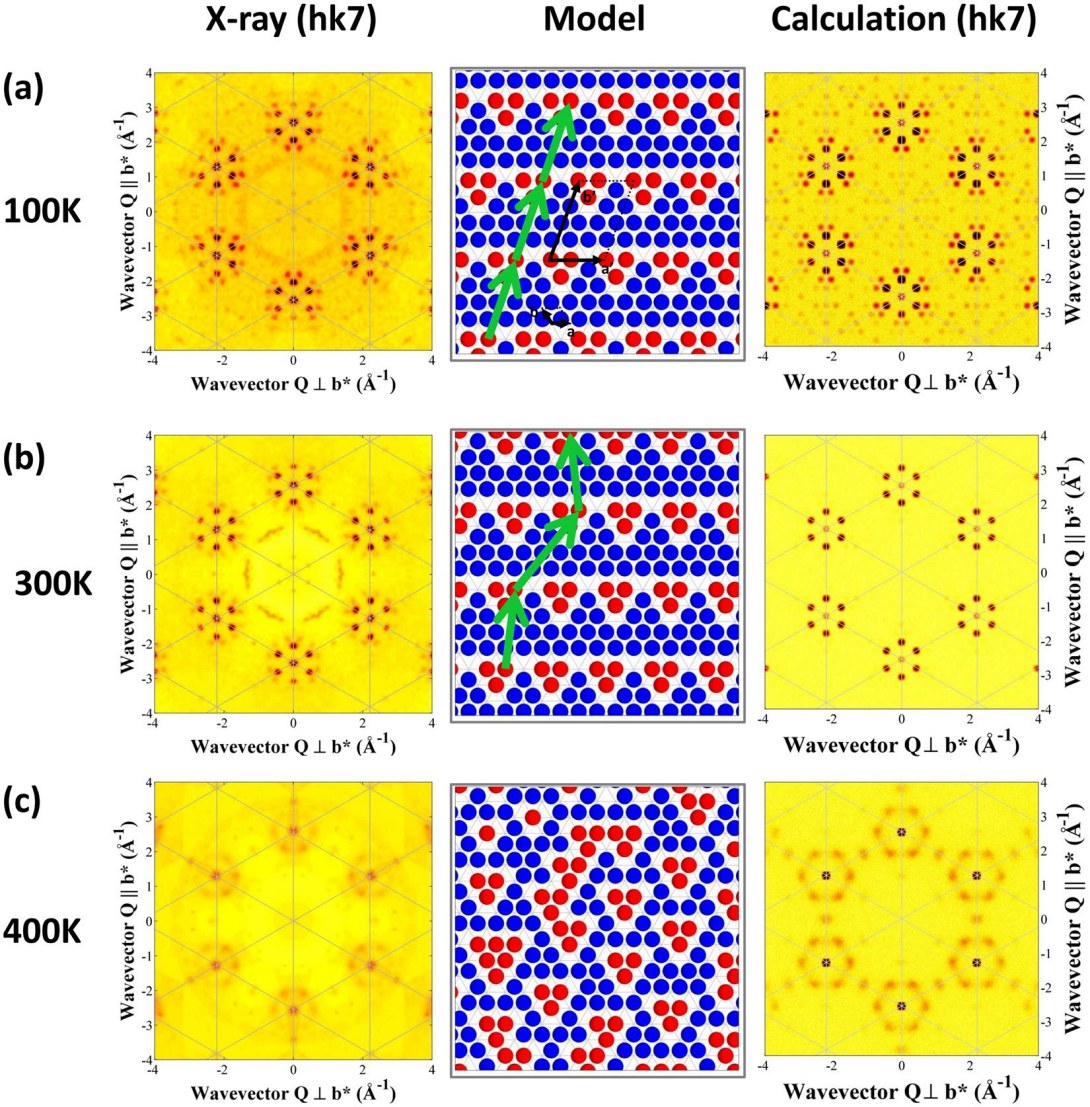
X-ray diffraction measurements of sodium ordering in Na_0.8_CoO_2_. (**a**) Long-range order in the fully ordered tri-vacancy stripe phase gives sharp superlattice reflections. A tri-vacancy cluster comprises three Na-ion vacancies and three Na ions promoted from 2*d* (blue) to 2*b* (red) sites. Hexagonal unit cell and supercell vectors are shown (black). Green vectors link equivalent sites in successive stripes. (**b**) In the partially disordered tri-vacancy stripe phase, the stripes are ordered long range, but the locations of the tri-vacancy clusters are not correlated between stripes, and there is a reduction in intensity of some of the higher order superlattice peaks. (**c**) In the disordered phase there are no sharp superlattice reflections, and the characteristic diffuse scattering pattern arises from short-range ordering of multi-vacancy clusters. The calculated intensities are from the supercell of the ordered tri-vacancy stripe phase^25^ (**a**), a section of crystal with long-range ordering of stripes, but with random translations of the tri-vacancy clusters within stripes (**b**), and from a Monte Carlo simulation of a disordered mixture of di-, tri- and quadri-vacancy clusters (**c**).

Single crystals of Na_0.8_CoO_2_ were grown using the optical floating-zone technique at Royal Holloway. The temperature dependence of the sodium ordering was determined using single-crystal x-ray diffraction. Figure 2 shows the scattering in the (*hk*7) plane, where the superstructure reflections and diffuse scattering patterns are particularly clear. The full *l*-dependence of the scattering is presented in Supplementary Figs 1–6. At low temperatures the sample adopted the fully ordered superstructure comprising stripes of tri-vacancy clusters, see Fig. 2(a). Above *T* ~ 290 K there is a Na reordering transition to the partially disordered stripe phase in which the positions of the stripes are ordered long range, but the positions of the tri-vacancies within stripes are not correlated between stripes^25^, see Fig. 2(b).

At *T* ~ 370 K the sharp superlattice reflections in x-ray diffraction experiments disappear, and anisotropic rings of diffuse scattering are observed around some reciprocal lattice points. Previous Monte Carlo simulations predicted a mixture of di-, tri- and quadri-vacancy clusters that do not order long range in the disordered phase^23^. Our simulations for *x* = 0.8 are in excellent agreement with the experimental data, see Fig. 2(c), and this diffuse scattering pattern is the signature of the disordered mixture of multi-vacancy clusters.

### Molecular dynamics simulations

*Ab initio* molecular dynamics simulations predict that the diffusion mechanism of Na_*x*_CoO_2_ in the high temperature disordered phase is highly sensitive to Na-Na correlations, and differs from the di-vacancy mechanism of Li_*x*_CoO_2_^30^, due to the stabilisation of multi-vacancy clusters comprising vacancies on 2*d* sites and ions promoted to 2*b* sites^31^. Figure 1(c) shows typical diffusion pathways.

We performed first-principles density-functional calculations and MD simulations incorporating the observed Na-Na correlations using the CASTEP code^32^. It is particularly instructive to examine the calculations in the partially disordered stripe phase at *T* ~ 350 K, since the relatively small departures from a simple superstructure make it a good prototype in which to understand self-diffusion in Na_*x*_CoO_2_.

**Figure 3:**
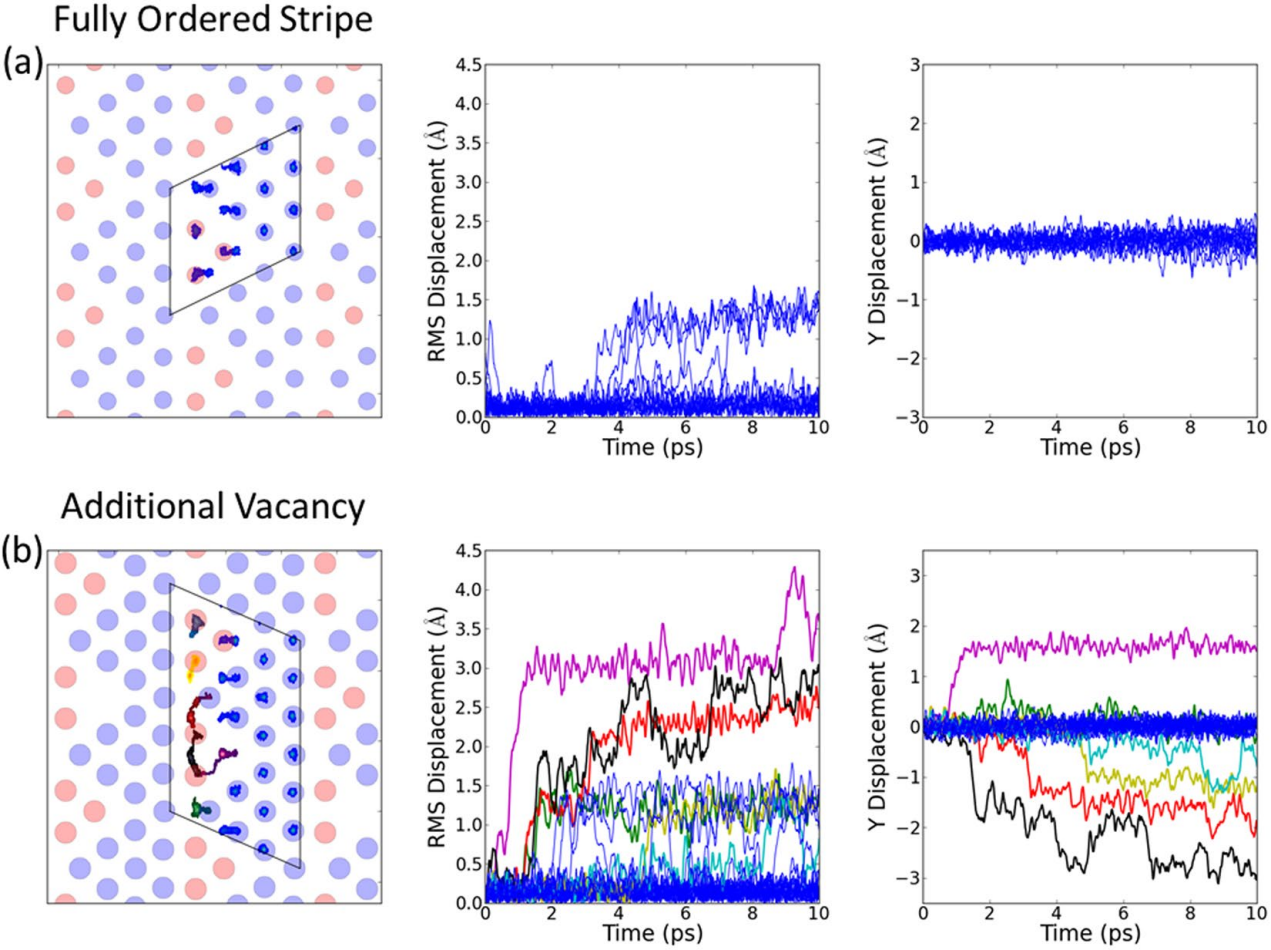
Molecular dynamics simulation of diffusion in Na_0.8_CoO_2_ in the stripe phase. The left-hand panel shows the trace of sodium positions over the duration of the simulations (the boxes show the cells used in the MD simulations), the middle panel shows the root-mean-square displacement of individual sodium ions from their initial sites as a function of time, and the right-hand panel shows displacements along the stripe direction. The different colours give trajectories for different ions, with the same colour for a given ion in successive panels from left to right. The simulations were performed at *T* ~ 350 K for the tri-vacancy stripe phase (**a**) fully ordered and (**b**) with an additional vacancy on a 2*b* site. For the ideal superstructure there are local hops back and forth between 2*d* and 2*b* sites perpendicular to the stripe, but there is no bulk self-diffusion. The introduction of additional disorder in the stripes results in multiple hops between 2*d* and 2*b* sites with components along the stripes. Chains of hops of different ions results in dynamic irreversibility and self-diffusion along the stripe.

**Figure 4:**
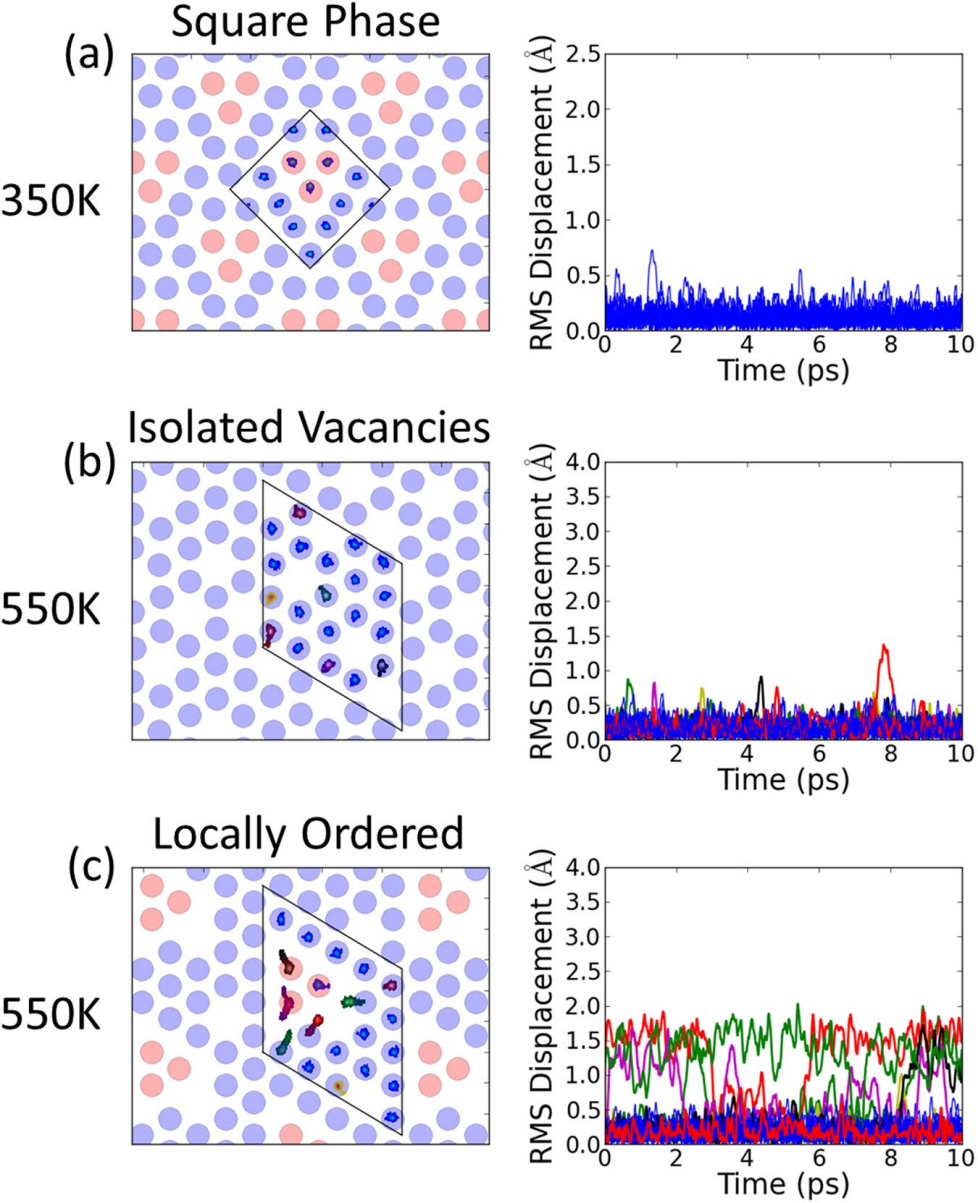
Molecular dynamics simulation of diffusion in Na_*x*_CoO_2_ in the square and disordered phases. The left-hand panels show the trace of sodium positions over the duration of the simulations (the boxes show the cells used in the MD simulations), and the right-hand panels show the root-mean-square displacement of sodium ions as a function of time. The different colours give trajectories for different ions, with the same colour for a given ion in successive panels from left to right. (**a**) For the square phase the tri-vacancy clusters are well separated, and there are very few hops compared to the stripe phase. (**b**) For isolated 2*d* vacancies there is negligible hopping of ions. (**c**) In the disordered phase the introduction of a vacancy next to a tri-vacancy cluster results in multiple hops. For interconnected pathways of the type shown in Fig. 2(c) bulk translational diffusion is predicted by consideration of the available pathways, though the timescale of these simulations is insufficiently long to demonstrate this directly.

Figure 3(a) shows the results from an ideal striped arrangement with no additional disorder. The interconnected tri-vacancy clusters result in much more hopping between 2*b* and 2*d* sites than for the separated tri-vacancy clusters in the square phase, see Fig. 4(a). For ideal stripes, ions are essentially only able to hop back and forth locally perpendicular to the stripes. This can result in the translation of tri-vacancy clusters along the stripes, but generates no net contribution to self-diffusion. Zero net diffusion is also expected for other ideal superstructures. Hence the correlation factor *f* = D*/*D*^*RW*^ ~ 0, where D* is the bulk self-diffusion coefficient, and *D*^*RW*^ is the microscopic random-walk diffusion coefficient.

The introduction of an additional vacancy on a 2*b* site results in hops between 2*b* and 2*d* sites with components along the stripe, see Fig. 3(b). In this case, individual ions exhibit a sequence of hops. Furthermore, the hops between different ions are correlated so that chains of hopping ions follow the initial hop towards a vacancy. This is expected to lead to dynamical irreversibility in the trajectories of the Na ions. At each stage in any chain, there are multiple possible hops, including perpendicular ones, so the likelihood of individual ions exactly retracing a particular path to the original starting configuration is negligibly small. Hence, it is reasonable to expect that *f* is significantly greater than zero.

In contrast, the addition of a 2*d* vacancy away from the stripes corresponds to the diffusion of isolated vacancies in the 2*d* lattice, and it is found to be negligible. This confirms that we really do have one dimensional diffusion channels along the stripes.

We have also performed simulations in the disordered phase at *T* ~ 550 K, and we again find that isolated vacancies on the 2*d* sites do not contribute towards diffusion, see Fig. 4(b). In contrast, vacancies next to multi-vacancy clusters lead to many 2*d* to 2*b* hops, see Fig. 4(c). The fact that local Na-Na correlations are important in lowering activation energies for Na-ion hops is in agreement with previous MD simulations in the disordered phase^31^. We have measured the actual short-range Na-Na correlations in this phase via diffuse x-ray scattering, and the pattern obtained from our MC simulations in Fig. 2(c) contains the interconnected pathways between multi-vacancy clusters required for bulk translational diffusion.

### Quasi-elastic neutron scattering experiment

Na has a significant incoherent neutron scattering cross section, and this scattering is related to the time-dependent self-correlation function. Hence the incoherent QENS enables both spatial and temporal aspects of the hopping mechanism of diffusing Na ions to be studied directly.

**Figure 5:**
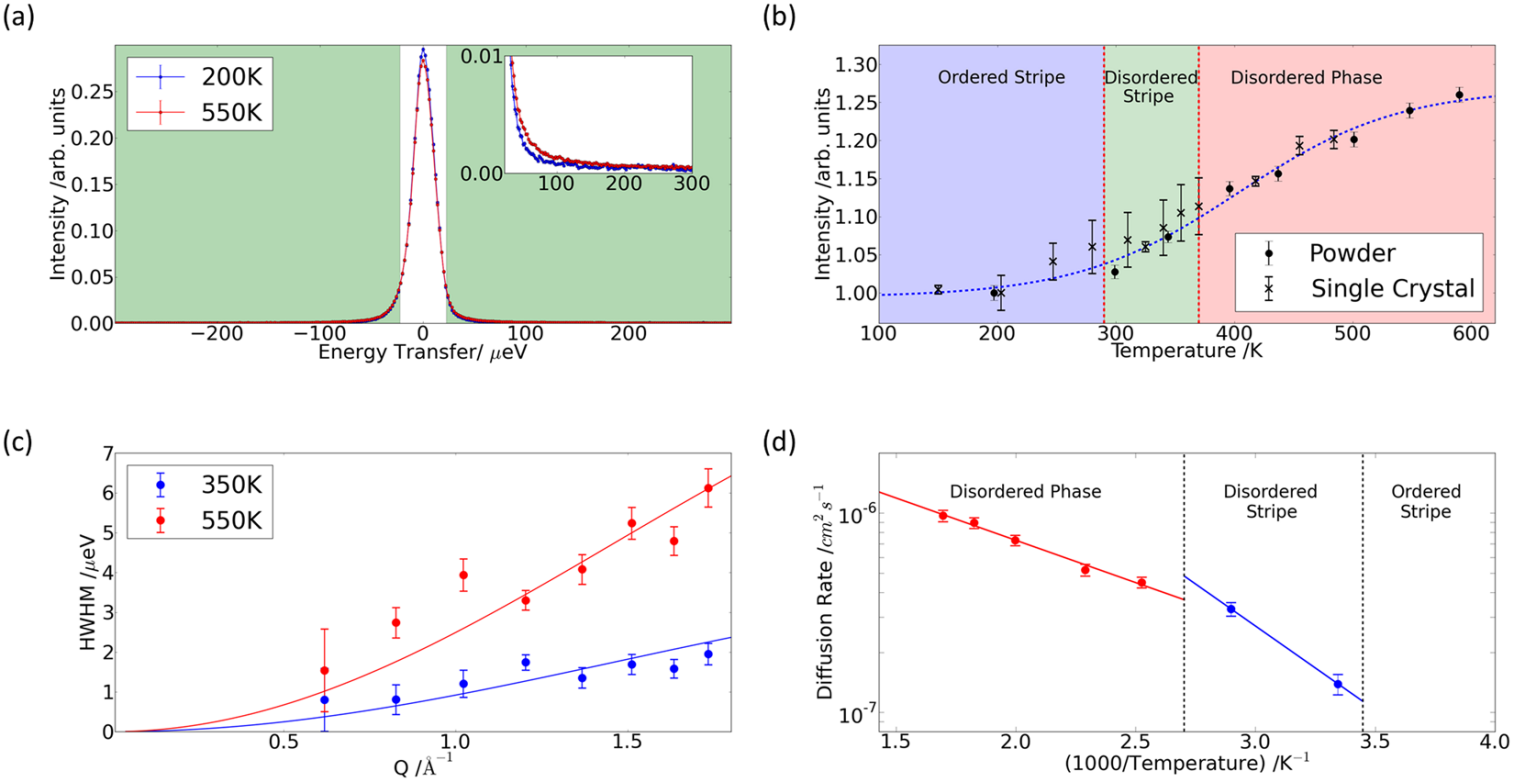
Quasi-elastic neutron scattering measurements of diffusion in Na_0.8_CoO_2_. (**a**) QENS spectra from Na_0.8_CoO_2_ at *T* ~ 200 K (blue) and *T* ~ 550 K (red) integrated over *Q*. At elevated temperature there is a transfer of intensity from the elastic region near *E* ~ 0 meV to the wings above |*E*| > 22.5 µeV (green shaded region). Inset shows increase in the quasi-elastic region. (**b**) QENS intensity integrated over *Q* and energy transfer as a function of temperature for the powder and single-crystal samples – the dotted line is a guide to the eye. There is an increase in QENS in the partially disordered stripe and disordered phase, and this is correlated with the onset of superionic diffusion by Na^29^. (**c**) QENS energy width as a function of *Q* in the fully disordered phase (red, *T* ~ 550 K) and the partially disordered stripe phase (blue, *T* ~ 350 K). Fits of the Chudley-Elliott model of jump diffusion for 2*d* − 2*b* hops are shown as solid lines. (**d**) Arrhenius plots of the diffusion coefficients from the fits of the Chudley-Elliott model to the QENS data in the disordered phase (red) and partially ordered stripe phase (blue) give activation energies *E*_A_ ~ 85(5) meV and 170(20) meV, respectively.

QENS measurements of Na-ion diffusion were performed on single-crystal and high-purity-powder samples using the OSIRIS spectrometer at ISIS^33^ with an energy resolution, Δ*E*_HWHM_ ~ 12.7 µeV. The data at *T* ~ 200 K were compared with the incoherent scattering from a vanadium standard sample, and at this temperature no energy broadening was detected. However, quasi-elastic energy broadening was detected at elevated temperatures. Figure 5(a) compares an energy scan at *T* ~ 550 K with the corresponding data at *T* ~ 200 K. There is a large, static incoherent background from the Co ions, but we were able to detect a quasi-elastic energy broadened component from dynamic incoherent scattering from Na ions at elevated temperatures. It is challenging to detect the quasi-elastic signal above the elastic background, but the expected dip in intensity at the elastic point and increase in intensity in the quasi-elastic wings is clear.

The component in the quasi-elastic wings integrated over all wave-vector transfers, *Q*, is presented in Fig. 5(b) as a function of temperature. The powder data are combined with data from a single-crystal sample of the same composition. There is a marked increase in quasi-elastic intensity in the partially disordered stripe and fully disordered phases, confirming the dynamic nature of the disorder in these phases. Hence the quasi-elastic energy broadening is detected where Na-ion diffusion is observed using bulk measurements^21^, and it is correlated with the onset of superionic diffusion reported previously from NMR studies^29^.

We were able to resolve the *Q* dependence of the energy line width of the QENS from the powdered sample, and this enabled us to determine the diffusion mechanism. The data from the single-crystal sample were consistent, but they were not used further due to large statistical errors. At all temperatures where energy broadening is detected the width tends to zero as *Q* tends to zero, as expected for translational diffusion. Furthermore, we see QENS intensity at all values of *Q* below *E* ~ 200 µeV, where there is no intensity from rattling modes^28^ and, therefore, we can rule out any significant contribution from localised motion.

Figure 5(c) compares the *Q* dependence of the energy line width with a fit of the Chudley-Elliott model of jump diffusion^34^, with the hopping distance, *d*_*QENS*_, fixed at the 2*d* to 2*b* separation. There is excellent agreement with the data at all temperatures, suggesting that 2*d* to 2*b* hops are the dominant diffusional process in both dynamic phases. The fit to the QENS data at *T* = 300 K gives a mean residence time, *τ*_*QENS*_ = 490(60) ps. The diffusion coefficient for a random walk in 1D along the stripe, *D*^*RW*^ = *d*_1*D*_^2^/(2*τ*_*1D*_) = *d*_*QENS*_^2^/4*τ*_*QENS*_ = 1.4(2) × 10^−7^ cm^2^ s^−1^, see Supplementary Figure S7. This corresponds to an upper limit.

The temperature dependence of the diffusion coefficients from the QENS data are shown in the Arrhenius plot in Fig. 5(d). Different activation energies are determined in the partially disordered stripe phase and the fully disordered phase. At elevated temperatures in the disordered phase our experimentally determined activation energy, *E*_A_ ~ 85(5) meV, is in good agreement with the value of about 100 meV obtained from previous MD simulations^31^. There is a moderate increase in activation energy to *E*_A_ ~ 170(20) meV upon entering the stripe phase, which is comparable to the values obtained in our transition-state searches for 2*d* to 2*b* hops perpendicular to the stripes, see Supplementary Figure S8. The activation energies for hops of Na vacancies along stripes are an order of magnitude higher.

## Discussion

**Figure 6:**
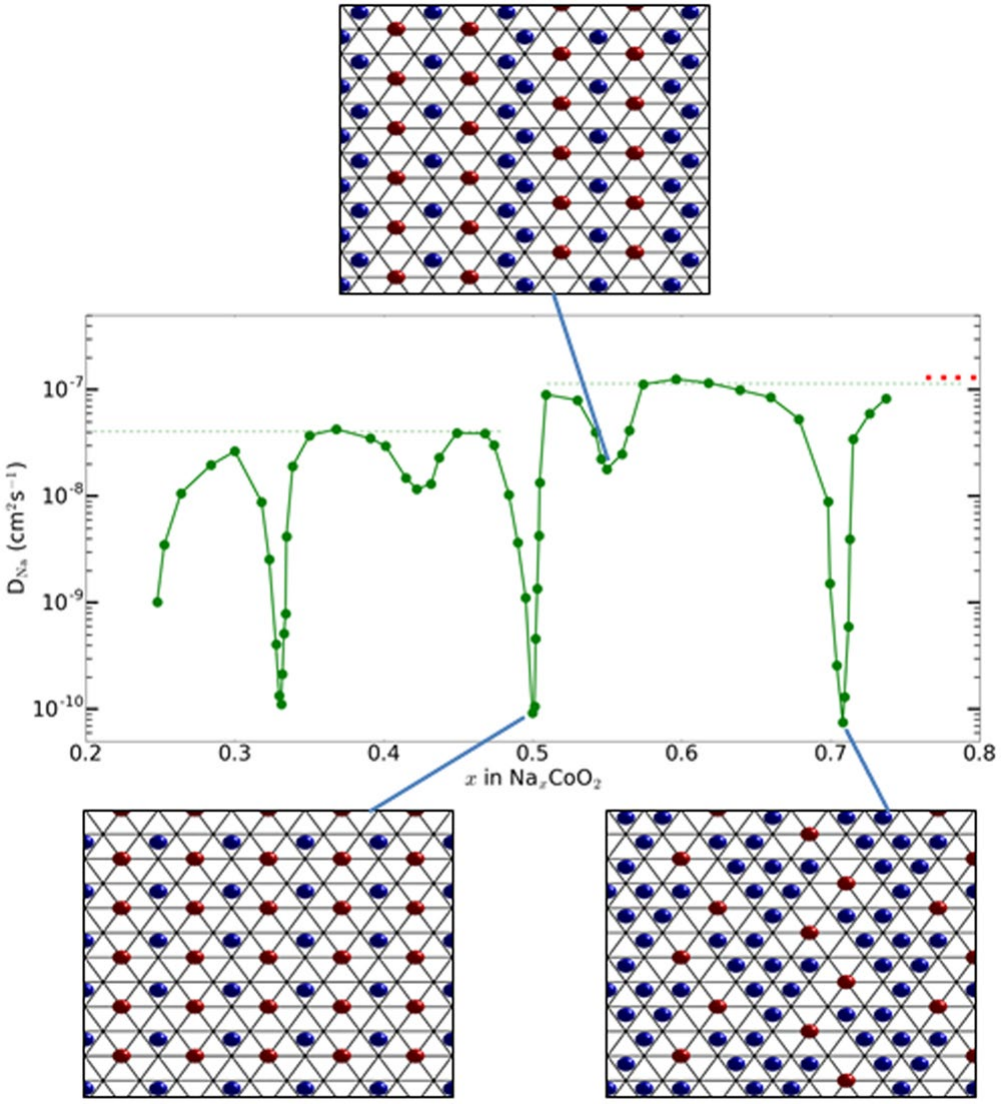
Bulk diffusion rate of Na_*x*_CoO_2_ as a function of sodium concentration, *x*. The measurements performed at ambient temperature using the Potentiostatic Intermittent Titration Technique (PITT) are shown as green circles. The diffusion rate determined at room temperature in our QENS study in the striped phase (Fig. 2(b)) and composition in the vicinity of *x* ~ 0.8 is shown as a red dashed line, and this value is comparable to the optimum rate for *x* > 0.5 (green dashed line). Sharp drops are observed at simple fractional values of *x* that correspond to Na-ion superstructures, and these structures are shown for *x* = 1/2, 5/9 and 5/7^24^. PITT data reproduced from Fig. 4(c) in ref.^21^.

Figure 6 shows the concentration, *x*, dependence of the self-diffusion coefficient, D*, of Na_*x*_CoO_2_ measured at room temperature using the potentiostatic intermittent titration technique (PITT) from ref.^21^. These PITT measurements show very sharp decreases in D* at values of *x* that correspond to ideal superstructures, consistent with our prediction that fully ordered superstructures have correlation factors *f* ~ 0. The concentration of our QENS sample is in the vicinity *x* ~ 0.8. At this precise composition one would expect a sharp decrease in D* due to the fact that *f* ~ 0 for the striped superstructure, see Fig. 3(a). However, at values of *x* sufficiently below this value one would expect additional vacancies, see Fig. 3(b), with *f* significantly above zero. Comparison of the random-walk diffusion coefficient *D*^*RW*^ obtained at room temperature by QENS with the rate obtained from PITT measurements away from ideal superstructures over the range *x* > 0.5, D* ~ 1.2(5) × 10^−7^ cm^2^ s^−1^, gives an empirical correlation factor comparable to unity, *f* = 0.9(4). Hence the microscopic QENS measurements are in excellent quantitative agreement with the bulk PITT measurements.

We now consider how the fundamental materials transport properties determined here relate to the operation of real batteries. PITT measurements using a single-crystal electrode of well-defined diffusion geometry and with small voltage steps^21^ gave outstanding agreement with our QENS measurements. However, there is a large discrepancy between these diffusion rates, *D*^*QENS*^ ~ *D*^*PITT*^ ~ 10^−7^ cm^2^ s^−1^, and the values extracted from a full cell using electrochemical impedance spectroscopy (EIS)^35^, *D*^*EIS*^ ~ 10^−10^ cm^2^ s^−1^, which has not been reconciled. We note that the galvanostatic cycles in ref.^35^ do not reproduce the clear-cut sodium reordering transitions reported previously in ref.^19^, suggesting that the measurements using EIS are far from equilibrium. It seems reasonable to speculate that the EIS sample contained a concentration gradient with a variable composition across the electrode. Hence regions of low mobility corresponding to ideal superstructures could limit the overall diffusion rate to *D*^*EIS*^ ~ 10^−10^ cm^2^ s^−1^. Other factors such as interface effects may play a role, but the PITT results at *x* = 0.5 and 0.71, *D*^*PITT*^ ~ 10^−10^ cm^2^ s^−1^, make this a particularly appealing explanation, see Fig. 6. Hence the low diffusion rate corresponding to ideal superstructures is likely to severely limit battery performance, particularly at high charge/discharge rates where electrodes are far from equilibrium.

A transformation from 1D to 2D Na ion diffusion has previously been predicted on the basis of Fourier difference maps from a powder neutron diffraction study of Na_0.7_CoO_2_^36^. The observation of two different in-plane lattice parameters, is consistent with the presence of stripe ordering. The diffraction results imply that Na ions hop only in one direction, and we show an example of how this can occur with a fully ordered superstructure.

In summary, we have determined the diffusion mechanism in the prototypical layered Na-ion battery material Na_*x*_CoO_2_. This approach can readily be extended to understand the diffusion mechanism in other Na-ion battery materials. We show factors that enhance the ionic conductivity for layered Na-ion cathode materials (interconnected multi-vacancy clusters) and suppress diffusion (long-range ordered superstructures). Hence it will be possible to design electrodes and solid electrolytes with better ionic conductivity. For example, random doping in the transition-metal layer may be expected to promote the presence of defect clusters, while preventing the formation of fully ordered superstructures. Lithium has a smaller, but significant incoherent neutron scattering cross section and, therefore, this approach using the QENS technique could also potentially be applied to better understand diffusion in Li-ion battery materials.

## Methods

### Experiment

Crystals of Na_0.8_CoO_2_ were grown using an optical floating-zone furnace at Royal Holloway. Sub-millimetre single crystals were cleaved from the boule, and the structure was determined using x-rays at the Mo K-edge using an in-house Agilent Xcalibur X-ray diffractometer. The sample was placed in a stream of nitrogen gas from a Cryojet temperature controller, and at each temperature, large volumes of reciprocal space were surveyed with a CCD detector. Diffraction measurements at *T* ~ 100 and 300 K produced superlattice reflections that corresponded to the fully ordered and partially disordered tri-vacancy stripe phases^25^, respectively. The low-temperature superstructure was obtained via a model-independent reverse Monte Carlo fit to the superlattice reflections. An elegant alternative model based on di-vacancy clusters and with different periodicity^37^ can be ruled out by the data for this composition. Furthermore, the formation of tri-vacancy clusters at high *x* is supported by first-principles computer simulations^24^.

The phase at intermediate temperature is the minimal Na reordering required to account for the changes to the superlattice peaks. In order to calculate the diffraction from the partially ordered stripes, we start with the fully ordered structure in Fig. 2(a). The tri-vacancy clusters can sit on three possible sites within the stripes. We use the model proposed in ref.^25^ where the ordering of stripes is long range but the ordering of tri-vacancy clusters within the stripes is not coherent from one stripe to the next. The translation vectors for this phase are ***a***′ = 5***b*** + (*y* − 1)***a*** and ***b***′ = 5***b* + **(*y* − 4)***a*** where *y* = 0, ± 1 at random. A section of this partially disordered structure is shown in Fig. 2(b). The hopping of ions within the stripe phase in Fig. 3 provides a ready explanation for this disorder within the stripes.

The sharp superlattice reflections had disappeared at *T* ~ 400 K, and diffuse rings were observed around some reciprocal lattice points. The short-range ordering was investigated using Monte Carlo simulations with the long-range Coulomb and short-range repulsion model^23^. The pair energy between Na ions is the sum of a strongly repulsive short-range part preventing electronic shells from overlapping *V*_*sr*_ ~ *A*exp[−*r*/*r*_0_] − *C*/*r*^6^ with *A* = 424 eV, *r*_0_ = 0.318 Å and *C* = 1.05 eV Å^6^ from the literature^38^ and a long-range Coulomb contribution *V*_*c*_ ~ *e*^2^/4*πεε*_0_*r*. At nearest- and next-nearest-neighbour distances, we obtain respectively *V*^*1*^_*sr*_ ~ 2:53 eV (considered infinite at room temperature) and *V*^2^_*sr*_ ~ 0:06 eV (we neglect *V*_*sr*_ at larger distances). We take the effective dielectric constant to be isotropic and use the value *ε* ~ 6. Long-range Coulomb interactions between all Co, O and Na ions on three-dimensional periodic lattices were summed with an accuracy of ten digits using a generalization of the Ewald method^39^. At finite temperature, Monte Carlo simulations were performed in the canonical ensemble for a cell comprising 2,400 Na sites.

Large crystals were screened using the neutron Laue diffractometer SXD at ISIS^40^. A 1.96 g single-grain, 4 × 0.8 × 0.4 cm^3^, was selected for QENS with the fully ordered tri-vacancy stripe phase at temperatures below *T* ~ 290 K. The single crystal was mounted on an aluminium plate using aluminium wire to secure it. A high purity powder sample was prepared by crushing polycrystalline boules using a mortar and pestle in a helium atmosphere to limit exposure to water in the atmosphere, and 18 g of powder were inserted into an aluminium canister with a 2.2 cm diameter.

QENS measurements were carried out using the OSIRIS indirect geometry time-of-flight neutron spectrometer at ISIS^33^. OSIRIS combines a long-wavelength powder diffractometer with a backscattering spectrometer. A narrow energy resolution of 12.7 μeV HWHM is achieved using the (002) reflection of the bank of pyrolytic graphite analysers. The PG(002) analysers scatter neutrons of final energy 1.84 meV along collimated paths into 42 ^3^He detectors below the sample. The analysers are arranged to give angular coverage of 11° < 2θ < 148° corresponding to a *Q*-range of 0.18–1.8 Å^−1^. Samples were mounted in a closed-cycle cryostat with a hot stage under vacuum.

It is challenging to detect the QENS signal from Na above the large static incoherent background from Co. In the case of the single-crystal, it was only possible to obtain good counting statistics by integrating over *Q*. For the much larger powder sample the better counting statistics allowed the *Q*-dependence of the QENS to be determined. QENS measurements of the powder sample were carried out for 12 hours at *T* ~ 200, 300, 350, 400, 450, 500, 550, and 600 K. At *T* ~ 200 K the QENS is resolution-limited in energy transfer by reference to measurements from a vanadium standard. At all other temperatures quasi-elastic energy broadening was detected.

The data were reduced using the Mantid software suite (version 3.5.0)^41^. The detectors were grouped into pairs and energy bins of 4 μeV were chosen to reduce statistical noise. A combination of the static resolution function from the *T* ~ 200 K data, and a convolution of the resolution function and a Lorentzian function, was used to fit the scans in energy transfer at each *Q*. Fits of the Chudley-Elliot model^34^ to the *Q*-dependence of the Lorentzian FWHM gave the diffusion rate at each temperature. The variation in diffusion rate with temperature provides the activation energy and diffusion constant.

### Theory

The density functional theory (DFT) simulations used the plane-wave pseudopotential approach and spin polarised Perdew-Burke-Ernzerhof^42^ generalized gradient approximation as implemented by the CASTEP code^43^. Vanderbilt-type ultrasoft pseudopotentials were generated within CASTEP. For Na and Co the C7 CASTEP library was employed, whereas a custom-made slightly softer pseudopotential with a core radius of 1.5 Å was used for O. The plane-wave cut off was set to 500 eV based on convergence tests using a primitive cell of NaCoO_2_. The metallic nature of the conduction bands was described using Gaussian broadening of width 0.1 eV. MD simulations were of the Born-Oppenheimer type with first order wavefunction projection.

The MD simulated cells were considered large enough to minimise periodic-image interactions if each cell parameter was larger than 10 Å. The fully ordered stripe and square phase supercells contain 114 ions and have a lattice parameter greater than 10 Å. However, for calculations with additional vacancies in the stripe phase it was necessary to create a supercell comprising 226 ions, and in the disordered phase 190 ions. The sampling of *k*-space was on a 3 × 3 × 3 grid, equivalent to a spacing of around 0.2 Å^−1^. MD simulations were run within the *NVT* ensemble using a Langevin thermostat^44^. The thermostat for the stripe and square supercell calculations was set at *T* ~ 350 K, whereas the thermostat for the disordered phase calculations was *T* ~ 550 K. A 5 fs time step was deemed suitable given that the highest energy phonons have a period of 52 fs^28^. After an initial equilibration period of 1 ps, each calculation ran for a total of 10 ps.

Transition-state searches for each type of hop were performed using the complete Linear Synchronous Transit – Quadratic Synchronous Transit method^45^. This finds the minimum energy barrier of the transition by adjusting the positions of all ions from a maximum to find the optimal saddle point. Candidate hops were selected based on the structural features as well as motion observed in our MD simulations. The initial and final ionic positions were refined by geometry optimisation using the LBFGS algorithm^46^. The activation energy is *E*_A_ = 94 meV for a hop from a 2*d* to a 2*b* site *perpendicular* to the stripe, and it is an order of magnitude larger *E*_A_ = 1,304 meV for a hop from a 2*b* to a vacant 2*b* site *parallel* to the stripe (see Supplementary Figure S8).

The x-ray datasets generated during and analysed during the current study are available from the corresponding author on reasonable request. The MD simulations and CIF files are available via Royal Holloway’s Figshare repository from doi:10.17637/rh.5692846 (MD simulations), doi:10.17637/rh.5692942 (fully ordered stripe phase), doi:10.17637/rh.5692963 (partially ordered stripe phase) and doi:10.17637/rh.5693002 (disordered phase). All raw neutron data and the associated metadata obtained as a result of access to ISIS, reside in the public domain, with ISIS acting as the custodian. The QENS data can be accessed from doi:10.5286/ISIS.E.24090568 (single crystal) and doi:10.5286/ISIS.E.49912250 (powder).

## Electronic supplementary material

Supplementary Figures
