# Supplementary material for: Diffusion mechanism in the sodium-ion battery material sodium cobaltate
Source: Sci Rep. 2018 Feb 16;8:3210. doi: 10.1038/s41598-018-21354-5 (PMC5816598; doi:10.1038/s41598-018-21354-5)
Supplement: Supplementary file 1 — Supplementary Figures [file 41598_2018_21354_MOESM1_ESM.pdf]

Diffusion mechanism in the sodium-ion battery material sodium cobaltate

T. J. Willis, D. G. Porter, D. J. Voneshen, S. Uthayakumar, F. Demmel, M. J Gutmann, M. Roger, K. Refson, J. P. Goff

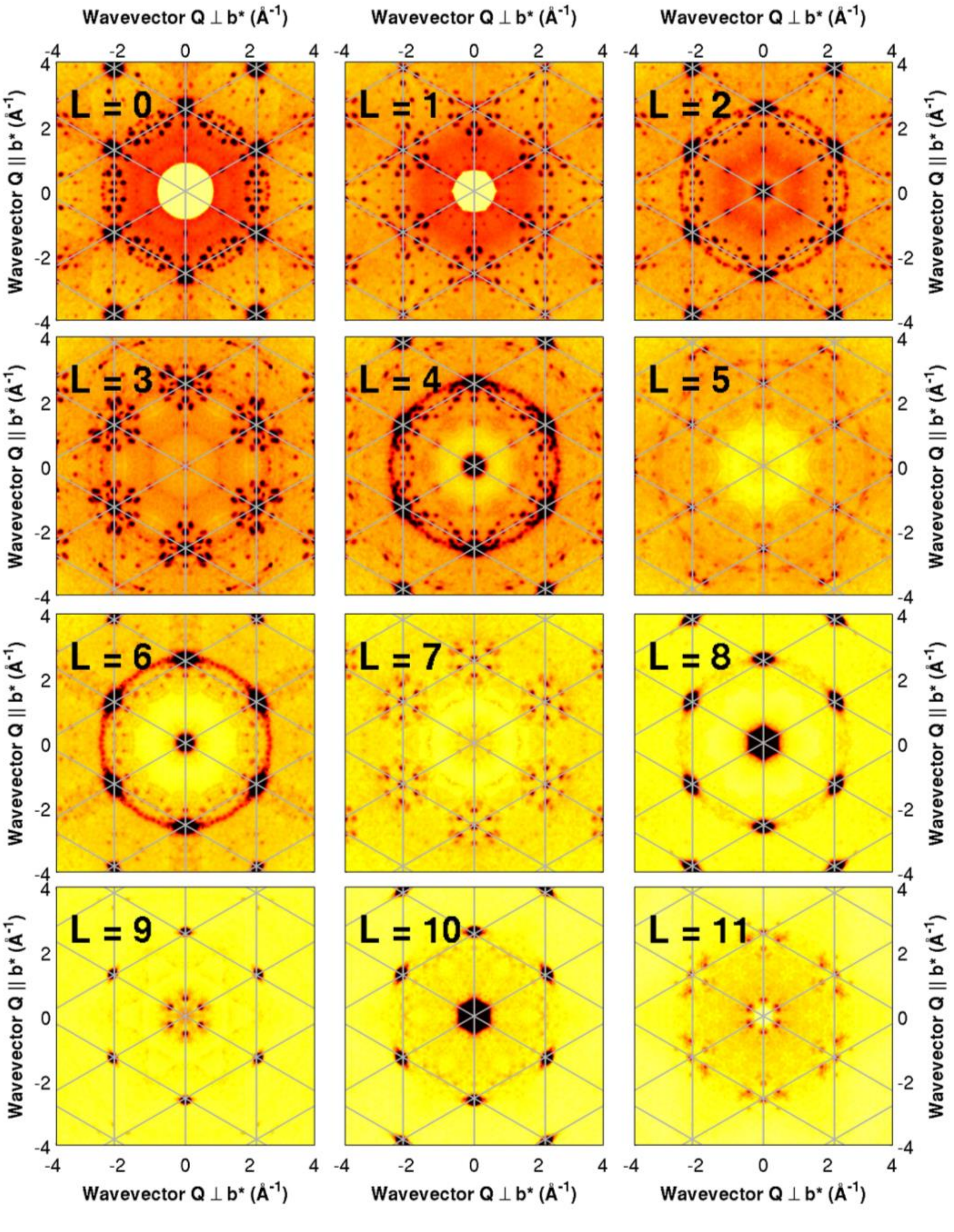

Fig. S1. Experimental x-ray diffraction data from $Na_{0.8}CoO_2$ at T ~ 100 K. Note there is some contamination from powder rings and from higher order ($\lambda/2$) reflections.

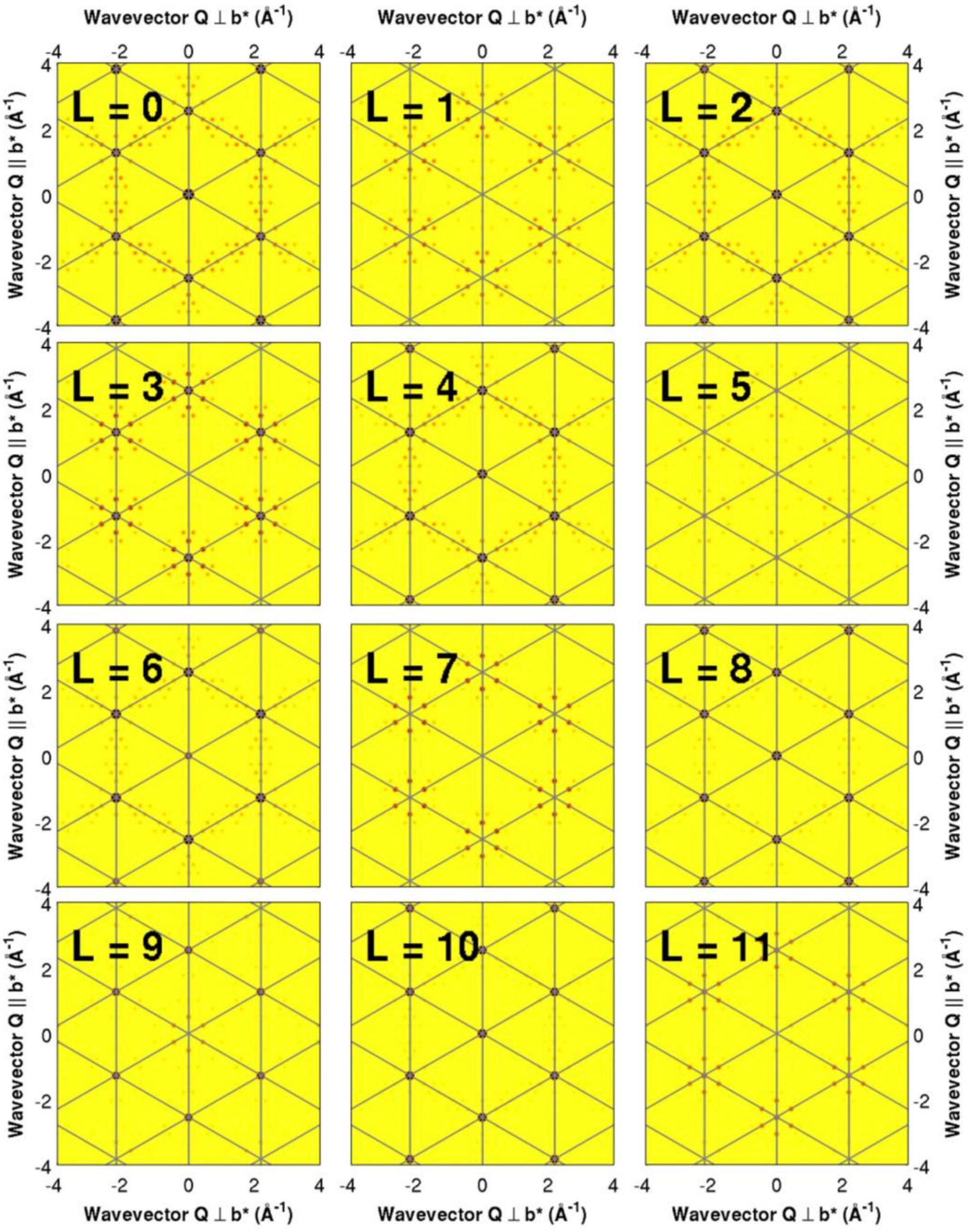

Fig. S2. Simulated x-ray diffraction pattern for $Na_{0.8}CoO_2$ at *T* ~ 100 K.

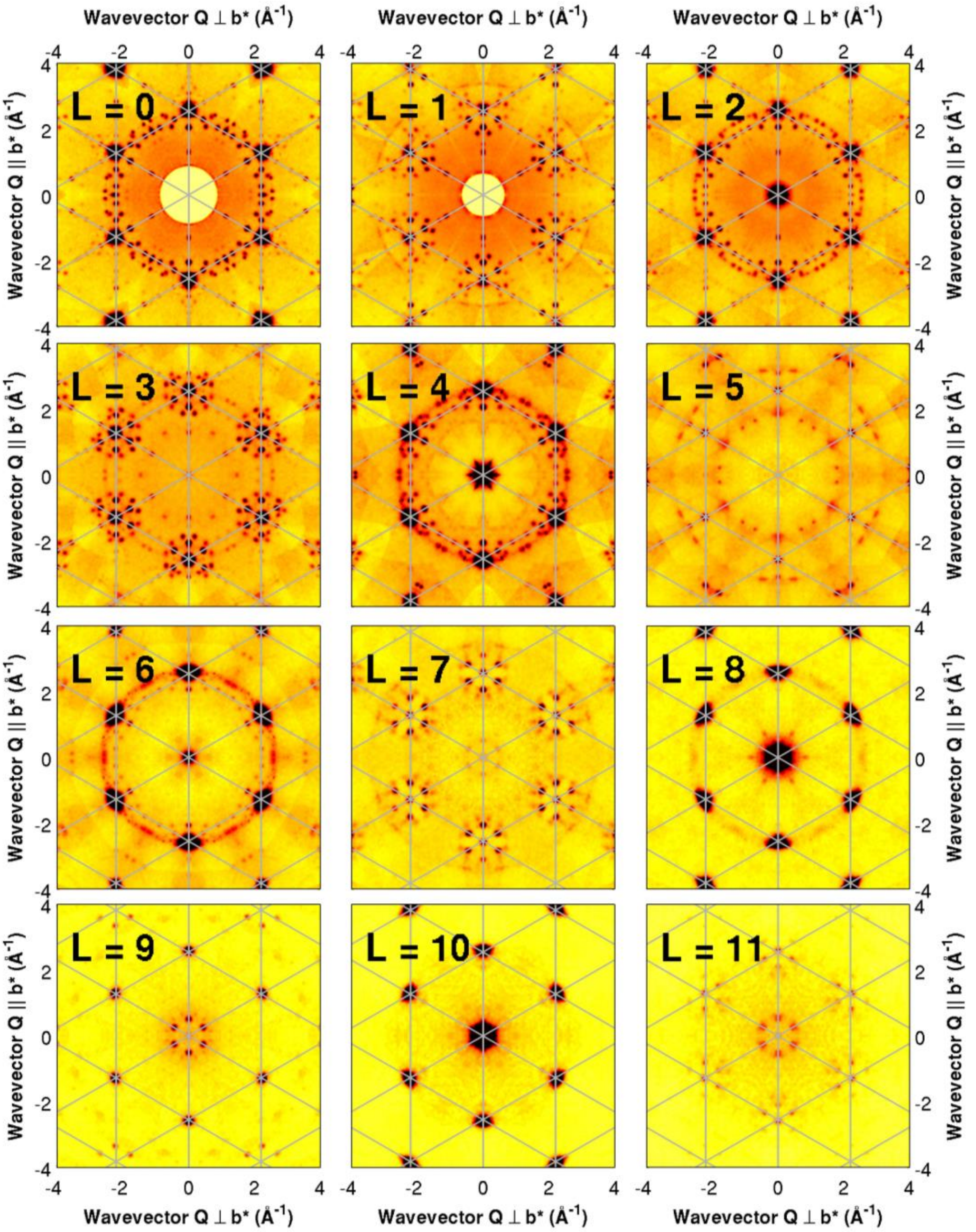

Fig. S3. Experimental x-ray diffraction data from $Na_{0.8}CoO_2$ at *T* ~ 300 K.

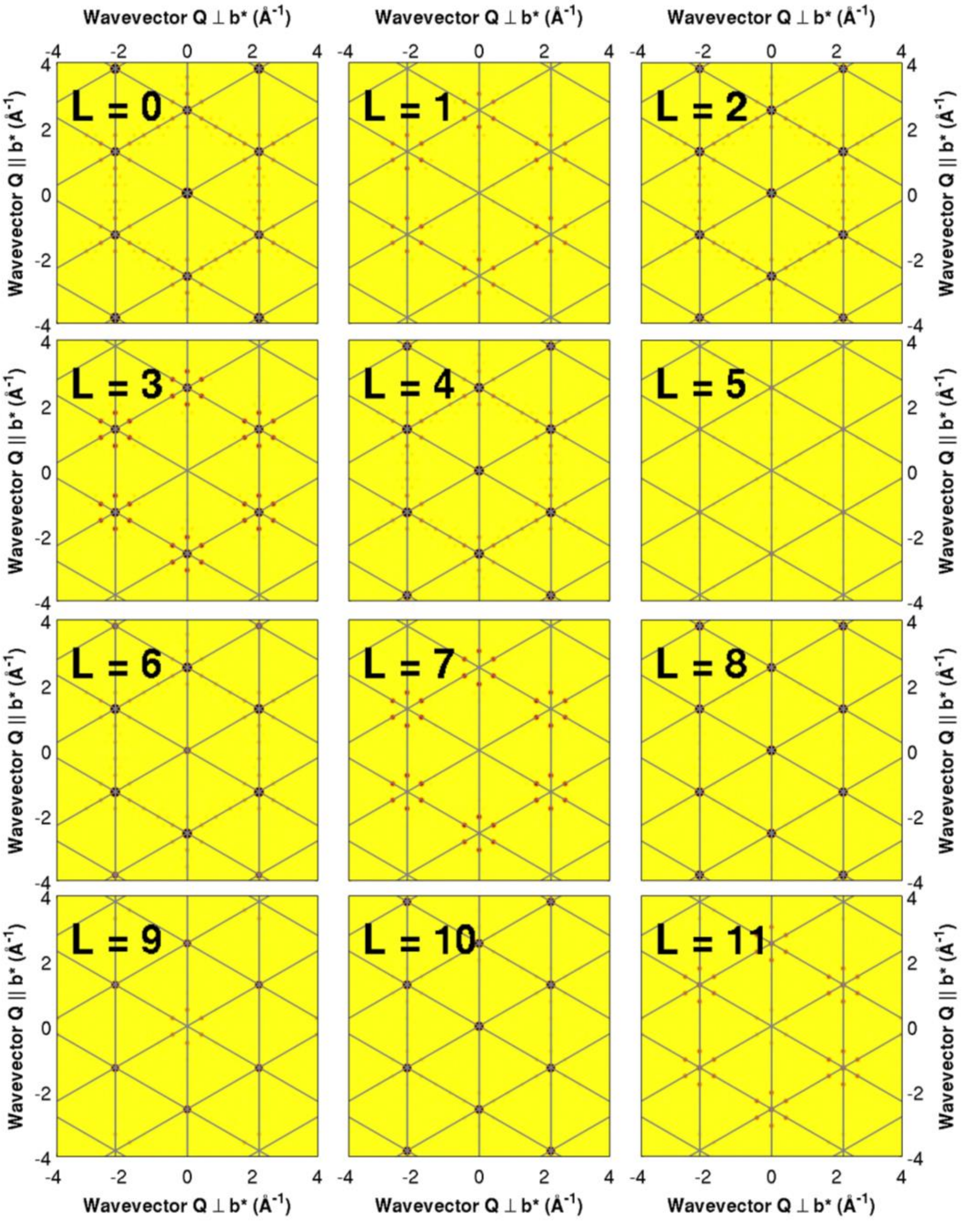

Fig. S4. Simulated x-ray diffraction pattern for $Na_{0.8}CoO_2$ at *T* ~ 300 K.

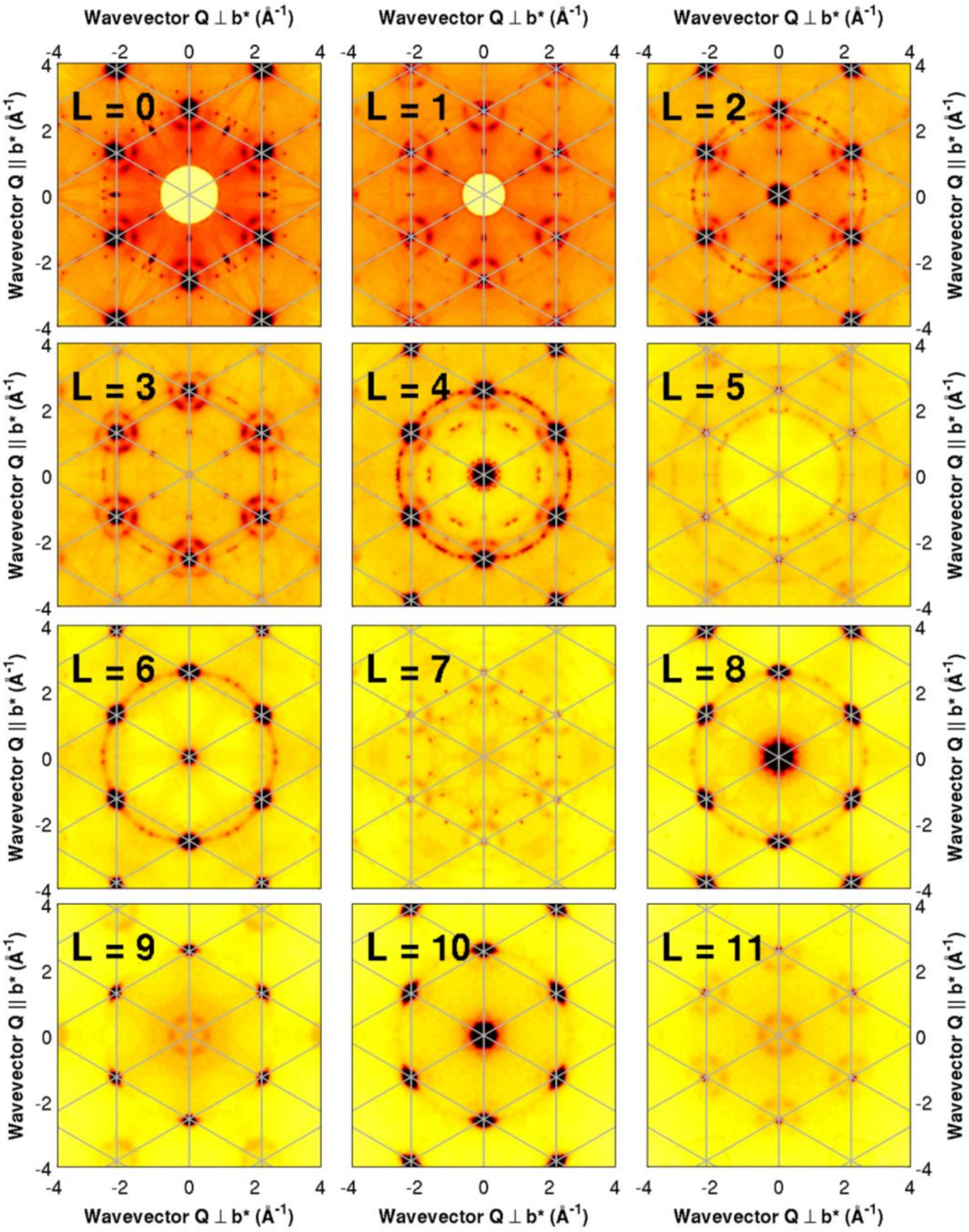

Fig. S5. Experimental x-ray diffraction data from $Na_{0.8}CoO_2$ at *T* ~ 400 K.

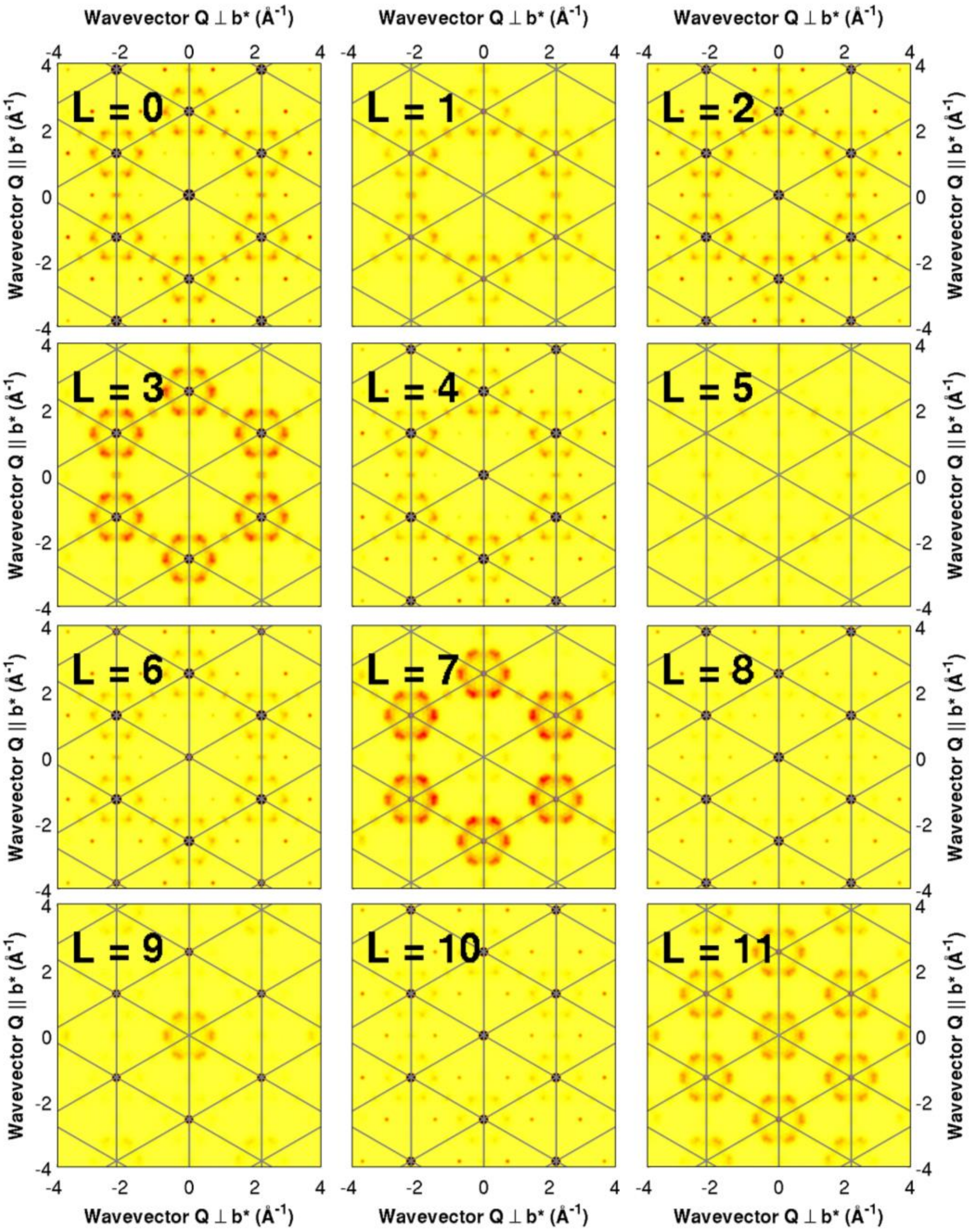

Fig. S6. Simulated x-ray diffraction pattern for $Na_{0.8}CoO_2$ at *T* ~ 400 K.

(a) 2D diffusion on a honeycomb lattice

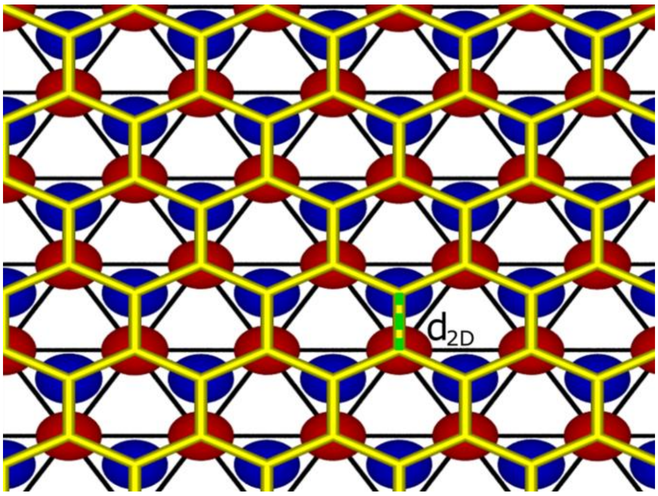

Hopping distance in plane:

$$d_{2D} = d_{QENS}$$

Mean residence time:

$$\tau_{2D} = \tau_{QENS}$$

Random-walk diffusion in 2D:

$$D_{2D} = \frac{d_{2D}^2}{4\tau_{2D}} = \frac{d_{QENS}^2}{4\tau_{QENS}}$$

(b) 1D diffusion along a stripe

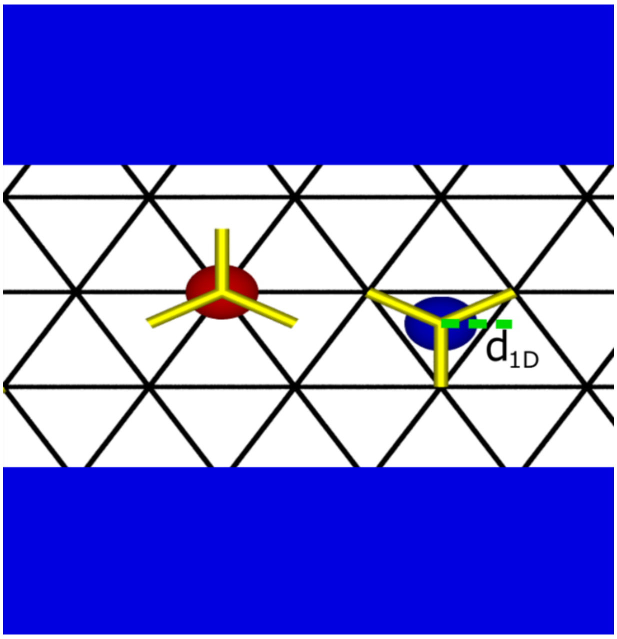

Hopping distance along stripe:

$$d_{1D} = \left(\frac{\sqrt{3}}{2}\right) d_{QENS}$$

Mean residence time:

$$\tau_{1D} = \frac{3}{2}\tau_{QENS}$$

Random-walk diffusion in 1D:

$$D_{1D} = \frac{d_{1D}^2}{2\tau_{1D}} = \frac{d_{QENS}^2}{4\tau_{QENS}}$$

Fig. S7. Random-walk diffusion coefficients. According to our MD simulations, the majority of Na ion hops are between 2*b* and 2*d* sites. (a) In the fully disordered phase Na ion hops are on a honeycomb lattice. (b) In the partially disordered striped phase, only the component of the hop along the stripe direction is relevant to self-diffusion. We assume that hops in any direction are equally likely, and this is supported by MD simulations with additional vacancies. In the striped phase, the residence time between hops with a component along the stripe is longer, since 1/3 of hops are perpendicular to the stripe and do not contribute towards the diffusion. The two resultant diffusion coefficients have the same dependence on the hopping distance, d_{QENS}, and residence time, τ_{QENS}, determined by QENS.

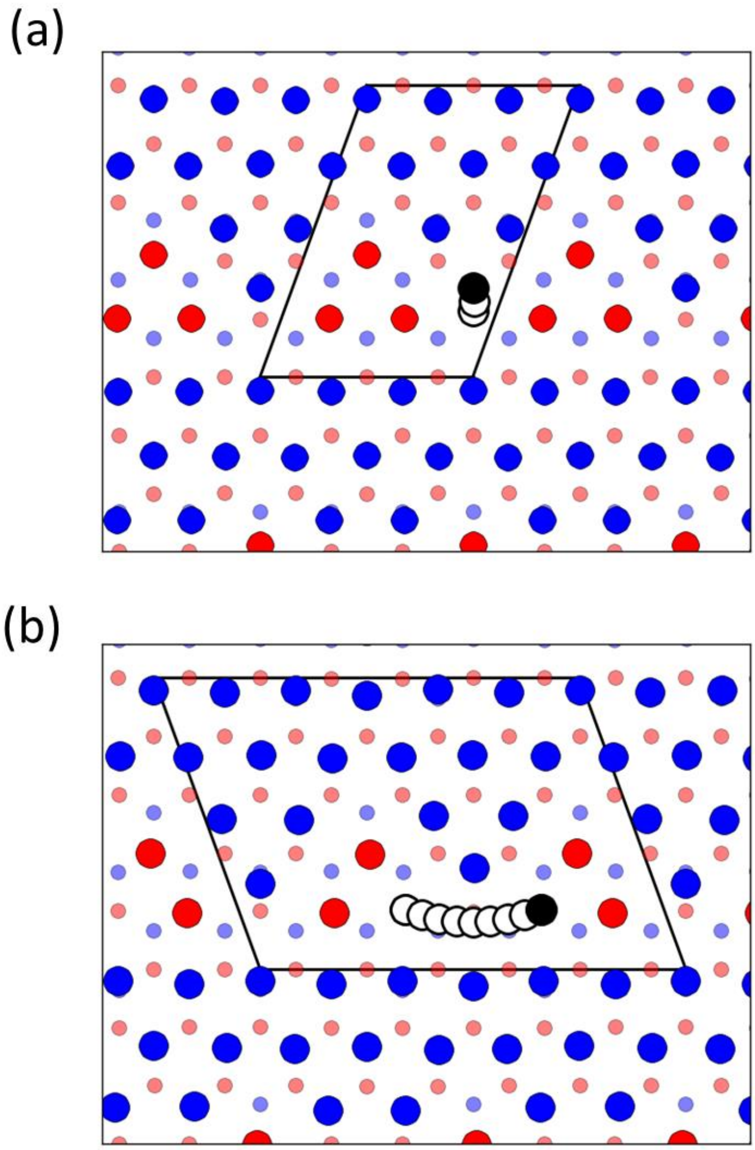

Fig. S8. Potential diffusion pathways in the stripe phase. (a) Hop from a 2*d* to a 2*b* site *perpendicular* to the stripe of tri-vacancy clusters. (b) Hop from 2*b* to a vacant 2*b* site *parallel* to the stripe. In this case the activation energy is an order of magnitude larger despite the presence of an additional vacancy, which might otherwise be expected to enhance diffusion. Red (blue) circles are Na 2*b* (2*d*) sites, large occupied and small vacant. Black circles show the initial position, and white circles represent successive positions of the diffusing ion. Only the initial, middle and final points are determined in the transition-state search.
